# INI1 mutations in meningiomas at a potential hotspot in exon 9

**DOI:** 10.1054/bjoc.2000.1583

**Published:** 2001-01

**Authors:** U Schmitz, W Mueller, M Weber, N Sévenet, O Delattre, A von Deimling

**Affiliations:** Department of Neuropathology, Charité, Humboldt University, Berlin, 13353, Germany; Laboratory of Molecular Cancer Pathology, Institut Curie, Paris, 75248, France

**Keywords:** INI1, SMARCB1, hSNF5, mutation, meningioma

## Abstract

Rhabdoid tumours have been shown to carry somatic mutations in the INI1 (SMARCB1/hSNF5) gene. A considerable fraction of these tumours exhibit allelic losses on chromosome 22. Allelic loss on 22q also is characteristic for meningiomas, however most of these alterations are considered to be associated with mutations of the NF2 gene. We examined a series of 126 meningiomas for alterations in the INI1 gene. Four identical somatic mutations in exon 9 were detected resulting in an exchange of Arg to His in position 377 of INI1. Our observations were reproduced both by using DNA from a new round of extraction and by employing overlapping primers. This mutational hotspot therefore appears to be an important target in the formation of a fraction of meningiomas. In addition, 4 novel polymorphisms of INI1 were characterized. Our data indicate that the INI1 is a second tumour suppressor gene on chromosome 22 that may be important for the genesis of meningiomas. © 2001 Cancer Research Campaign http://www.bjcancer.com


British Journal of Cancer (2001) 84(2), 199–201
© 2001 Cancer Research Campaign
doi: 10.1054/ bjoc.2000.1583, available online at http://www.idealibrary.com on IDEAL®

http://www.bjcancer.com

Short Communication

### INI1 mutations in meningiomas at a potential hotspot in exon 9

**U Schmitz[1], W Mueller[1], M Weber[1], N Sévenet[2], O Delattre[2] and A von Deimling[1]**

[1]Department of Neuropathology, Charité, Humboldt University, 13353 Berlin, Germany, [2]Laboratory of Molecular Cancer Pathology, Institut Curie, 75248 Paris, France

**Summary** Rhabdoid tumours have been shown to carry somatic mutations in the INI1 (SMARCB1/hSNF5) gene. A considerable fraction of these tumours exhibit allelic losses on chromosome 22. Allelic loss on 22q also is characteristic for meningiomas, however most of these alterations are considered to be associated with mutations of the NF2 gene. We examined a series of 126 meningiomas for alterations in the INI1 gene. Four identical somatic mutations in exon 9 were detected resulting in an exchange of Arg to His in position 377 of INI1. Our observations were reproduced both by using DNA from a new round of extraction and by employing overlapping primers. This mutational hotspot therefore appears to be an important target in the formation of a fraction of meningiomas. In addition, 4 novel polymorphisms of INI1 were characterized. Our data indicate that the INI1 is a second tumour suppressor gene on chromosome 22 that may be important for the genesis of meningiomas. © 2001 Cancer Research Campaign http://www.bjcancer.com

**Keywords**: INI1; SMARCB1; hSNF5; mutation; meningioma

Mutations in oncogenes and tumour suppressor genes contribute to the transformation of tumour cells. Frequently, these mutations are associated with allelic loss in the corresponding other parental region. Since allelic loss is readily detected such analyses can serve to point towards candidate genes residing in the affected regions. Recently, the INI1 (SMARCB1/hSNF5) gene, localizing to chromosome 22, was found to be mutated in paediatric malignant rhabdoid tumours (Versteege et al, 1998; Rousseau-Merck et al, 1999) and with a lower frequency in CNS tumours (Sevenet et al, 1999). INI1 therefore is an interesting candidate gene for tumours with allelic loss on chromosome 22 (LOH 22) seen in many different brain tumours. These include meningiomas (Dumanski et al, 1990), schwannomas (Seizinger et al, 1986), astrocytomas (James et al, 1988), ependymomas (James et al, 1990; Ebert et al, 1999) and glioblastomas (James et al, 1988). However, a major proportion of LOH 22 is associated with mutations in the NF2 gene on 22q12. This has been shown most evidently for meningiomas and schwannomas (Jacoby et al, 1994; Ruttledge et al, 1994a; Wellenreuther et al, 1995). On the other hand, the frequency of LOH 22 exceeds that of NF2 mutations and deletion mapping has revealed interstitial deletions not including the NF2 locus in some meningiomas (Ruttledge et al, 1994b). Therefore, additional meningioma genes have been postulated. An interesting candidate forwarded was β-adaptin, showing a reduced expression in meningiomas (Peyrard et al, 1994). However, a mutational analysis failed to detect mutations of β-adaptin leaving the relevance of its reduced expression unresolved (Peyrard et al, 1996).

In order to evaluate the role of the INI1 gene in the pathogenesis of meningiomas we analysed a series of 21 tumours with LOH 22 but without recognizable NF2 mutations and a second unselected series of 105 meningiomas.

*Received 10 April 2000*
*Revised 4 October 2000*
*Accepted 13 October 2000*

*Correspondence to:* A von Deimling

### MATERIAL AND METHODS

### Tumour specimens, histopathology and control DNA

Native tumour specimens and corresponding blood samples were obtained from patients treated at the University Hospital Bonn between 1990 and 1998. All tumours were classified according to the WHO guidelines (Kleihues and WK, 2000). The tumour specimens were examined microscopically prior to phenolic DNA extraction to exclude contamination by adjacent tissue. The analyses were performed on an initial series of 21 meningioma samples with LOH 22 but without NF2 gene mutations and on a series of 105 meningiomas without prior knowledge of either LOH 22 or mutations of NF2. All patients have consented to molecular analysis of their respective meningioma and constitutional DNA.

### SSCP analysis and direct sequencing

For analysis of the INI1 gene a set of previously published primers was employed (Versteege et al, 1998). The sequence for the newly devised overlapping PCR product of exon 9 was ex9f 5′-GAGA-GAAGGCTGGGTCTGAC and ex9r 5′-GTGCTGATGGGCTG-GTTACC. PCR was performed in a final volume of 10 µl containing 10 ng of DNA, 50 mM KCl, 20 mM Tris-HCl (pH 8.4), 200 µM of each dNTP, 0.1% gelatine, 10 pmol of each primer, 1.0 to 2.0 mM $MgCl_2$ and 0.25 U Taq polymerase (AmpliTaq® DNA Polymerase, Perkin Elmer, USA). Initial denaturation at 94˚C for 3 min was followed by 30 cycles on an automated thermal cycler (Biometra UNO Thermoblock, Göttingen, Germany). These included denaturation at 94˚C for 40 s, annealing at temperatures ranging from 50–62˚C depending on the primer pair for 40 s, and extension at 72˚C for 40 s. A final extension step at 72˚C for 10 min was added. Single strand conformation polymorphism

(SSCP) analysis was performed on a sequencing apparatus (BlueSeq 400, Boehringer Ingelheim, Germany) using 8%, 10%, 12% and 14% acrylamide gels. Electrophoresis was run at 2 W to 6 W and variable temperatures for 15 h. Silver staining of the gels was performed as previously described (von Deimling et al, 1993). Aberrantly migrating SSCP bands were excised and the DNA was extracted followed by reamplification with the same set of primers and sequencing on a semiautomatic sequencer (Applied Biosystems, model 377) using the BigDye Terminator Cycle Sequencing kit (Applied Biosystems). Each amplicon was sequenced bidirectionally.

### RESULTS AND DISCUSSION

4 of 126 meningiomas (3%) carried an identical somatic mutation in exon 9 of the INI1 gene. Corresponding DNAs from peripheral blood cells exhibited wild type status in all 4 patients (Figure 1, upper panel). The alteration was characterized by an G to A transition in the nucleotide 1130 of the coding sequence resulting in an missense mutation of Arg to His in codon 377 (Figure 1, lower panel). This nucleotide exchange was not observed in DNA samples from 104 healthy individuals, from 200 other intracranial brain tumours (A. v. D. unpublished data) and has not been reported previously. In order to exclude accidental contamination of template DNA with a potentially single mutated DNA amplicon, two independent strategies were employed. The DNA extraction was repeated from frozen tissue of these 4 patients. Subsequent SSCP analysis of exon 9 of INI1 provided identical results to those from our first round of experiments. Amplification of the polymorphic microsatellite D1S1608 using these DNAs as template yielded different alleles thereby confirming independent origin of the tissues. In addition, a novel primer pair was synthesized extending beyond the initially amplified fragment. PCR with these oligonucleotides yielded aberrantly migrating signals in the tumour DNA from the same patients. This PCR product could not have been amplified from a partly overlapping contaminating fragment. Thus, our results suggest a mutational hotspot at nucleotide 1130 of INI1 affected in approximately 3% of meningioma patients. One mutation occurred in our first series with 21 meningiomas exhibiting LOH 22 but without detectable NF2 mutations. Three additional mutations were detected in our second series containing 105

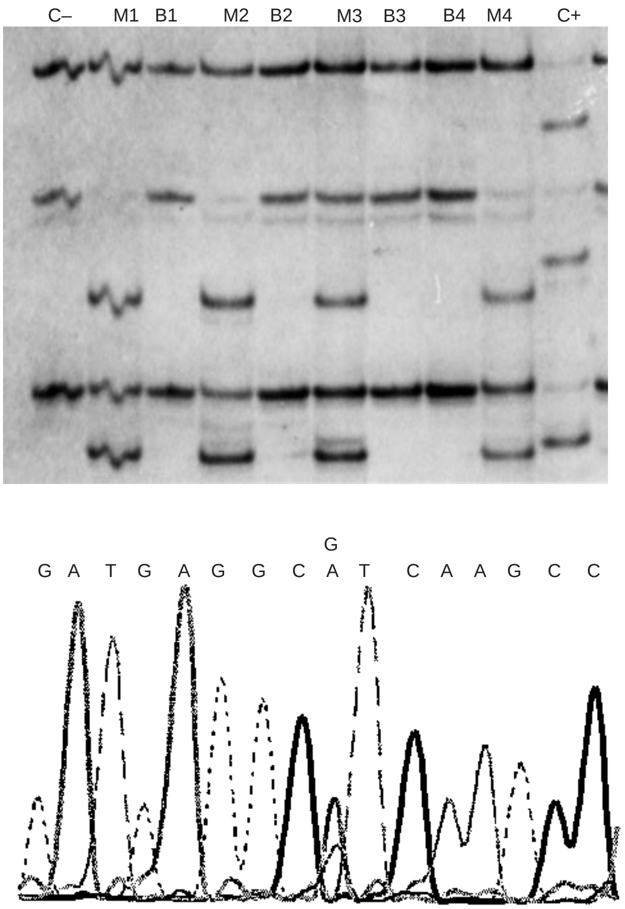

**Figure 1** Upper panel: SSCP of exon 9 of the INI1 gene in human meningiomas. Aberrantly migrating products are seen in exon 9 amplicons from meningioma DNA but not in amplicons derived from DNA of corresponding blood samples. C–= unaffected control, M =meningioma, B = peripheral blood leukocytes, C+ = positive control. Lower panel: Sequencing revealed a G to A transition in the nucleotide 1130 of the coding sequence resulting in an missense mutation of Arg to His in codon 377 in all four instances

**Table 1** Sequence polymorphisms in the INI1 gene

| Allele | Alleles in controls n f | Allele in patients n f | Position | Nucleotide | Amino acid |
|---|---|---|---|---|---|
| A1 | 78 – | 250 0.992 | exon 4 | 406 T | 136 Ser |
| A2 | 0 – | 2 0.008 | | 406 C | 136 Pro |
| B1 | 152 – | 251 0.996 | intron 5 | 628 + 13 c | |
| B2 | 0 – | 1 0.004 | | 628 + 13 t | |
| C1 | 84 0.609 | 154 0.611 | intron 5 | 629 – 58 c | |
| C2 | 54 0.391 | 98 0.389 | | 629 – 58 a | |
| D1 | 113 0.819 | 206 0.817 | intron 5 | 629 – 62 a | |
| | | | | 629 - (125 to 130) ccccc | |
| D2 | 25 0.181 | 46 0.183 | | 629 – 62 g | |
| | | | | 629 - (125 to 130) del c | |
| E1 | 188 0.783 | 223 0.885 | exon 7 | 897 G | 299 Ser |
| E2 | 52 0.217 | 29 0.115 | | 897 A | 299 Ser |
| F1 | 170 0.817 | 194 0.770 | intron 8 | 1119 – 41 g | |
| F2 | 38 0.183 | 58 0.230 | | 1119 – 41a | |

Allele frequencies, nucleotides affected and amino acid exchange are given for the six observed polymorphisms.

 *© 2001 Cancer Research Campaign*

meningiomas. Three of the four meningiomas contained different NF2 mutations each presumably resulting in premature chain termination. Two of those exhibited LOH 22 and one was not informative for the markers tested. The meningioma apparently wild type for NF2 exhibited LOH 22. These findings do not support the concept of INI1 mutations serving as an alternate mechanism to NF2 mutations in the pathogenesis of meningiomas. In contrast, the data rather indicate, that silencing of INI1 may co-operate with impairment of NF2 function. Histopathological evaluation of those 4 meningiomas with INI1 mutations revealed two meningiomas of the transitional and one meningioma each of the fibroblastic and meningotheliomatous subtype. A recent study analysed a series of 41 meningiomas for mutations of INI1 (Bruder et al, 1999). While this study examined 90% of the open reading frame of INI1, exons 1 and 9 were not included in this analyses, providing an explanation for missing the present observation.

We detected 4 novel polymorphisms in coding and intronic sequences of INI1. In addition we saw two previously described polymorphism in exon 7 and intron 8 (Bruder et al, 1999; Mine et al, 1999). A known variant in exon 6 was either not represented in our series or missed by the SSCP assay (Bruder et al, 1999). The variants were designated A–F with two alleles each. The C2 variant always combined a nucleotide exchange 58 base pairs upstream of exon 6 with a deletion of a single cytosine 100 base pairs upstream of exon 6. The allele frequencies in meningioma patients and healthy control individuals are given in Table 1. With the exception of allele E, the incidences of the respective alleles were identical in meningioma patients and in healthy control patients suggesting no modifying role of these INI1 variants in the pathogenesis of meningiomas. The frequency of E2 in our meningioma series was similar to the one described previously (Bruder et al, 1999), however, in healthy controls allele E2 was observed more frequently ($P < 0.01$). This difference should be interpreted with caution but warrants examination of an independent series.

INI1 is part of the SWI/SNF complex participating in transcriptional regulation by remodelling chromatin in an ATP dependent manner. INI1 contains three regions highly conserved among related proteins. These consist of two imperfect repeats, Rpt1 and Rpt2 and an c-terminal putative coiled coil domain (Morozov et al, 1998). Rpt1 is required for interaction with c-myc (Cheng et al, 1999). The c-terminal coiled coil domain is likely to be involved in protein-protein interactions yet to be specified. The somatic Arg to His mutations in codon 377 falls within the highly conserved coiled coil domain, and may therefore interfere with normal protein–protein interactions. However, no functional data on the effect of the Arg to His mutation in codon 377 are available.

In conclusion, we detected a hotspot mutation in the INI1 gene in 3% of the present series of meningiomas. This observation suggest, that INI1 is a second tumour suppressor gene on chromosome 22 that may be involved in the pathogenesis of meningiomas.

### ACKNOWLEDGEMENTS

Supported by the Deutsche Forschungsgemeinschaft (SFB 507).

### REFERENCES

Bruder CE, Dumanski JP and Kedra D (1999) The mouse ortholog of the human SMARCB1 gene encodes two splice forms. *Biochem Biophys Res Commun* **257**: 886–890

Cheng SWG, Davies KP, Yung E, Beltran RJ, Yu J and Kalpana GV (1999) c-MYC interacts with INI1/hSNF5 and requires the SWI/SNF complex for transactivation function. *Nat Genet* **22**: 102–105

Dumanski JP, Rouleau GA, Nordeskjoeld M and Collins VP (1990) Molecular genetic analysis of chromosome 22 in 81 cases of meningioma. *Cancer Research* **50**: 5863–5867

Ebert C, von Haken M, Meyer-Puttlitz B, Wiestler OD, Reifenberger G, Pietsch T and von Deimling A (1999) Molecular genetic analysis of ependymal tumors: NF2 mutations and chromosome 22q loss occur preferentially in intramedullary spinal ependymomas. *American Journal of Pathology* **155**: 627–632

Jacoby LB, MacCollin M, Louis DN, Mohney T, Rubio MP, Pulaski K, Trofatter JA, Kley N, Seizinger B, Ramesh V and Gusella JF (1994) Exon scanning for mutation of the NF2 gene in schwannomas. *Hum Mol Genet* **3**: 413–419

James CD, Carlblom E, Dumanski JP, Hansen M, Nordenskjold M, Collins VP and Cavenee WK (1988) Clonal genomic alterations in glioma malignancy stages. *Cancer Research* **48**: 5546–5551

James CD, He J, Carlbom E, Mikkelsen T, Ridderheim P-A, Cavenee WK and Collins VP (1990) Loss of genetic information in central nervous system tumors common to children and young adults. *Genes, Chromosomes and Cancer* **2**: 94–102

Kleihues P and WKC (2000) *Pathology and genetics of tumours of the nervous system* World Health Organization Classification of Tumours. IARC Press: Lyon

Mine N, Bando K, Utada Y, Nagai H, Araki T and Emi M (1999) Two single nucleotide polymorphisms of the hSNF5/INI1 gene. *J Hum Genet* **44**: 354–355

Morozov A, Yung E and Kalpana GV (1998) Structure-function analysis of integrase interactor 1/hSNF5L1 reveals differential properties of two repeat motifs present in the highly conserved region. *Proc Natl Acad Sci USA* **95**: 1120–1125

Peyrard M, Fransson I, Xie Y-G, Han F-Y, Ruttledge MH, Swahn S, Collins JE, Dunham I, Collins VP and Dumanski JP (1994) Characterization of a new member of the human beta-adaptin gene family from chromosome 22q12, a candidate meningioma gene. *Hum Mol Genet* **3**: 1393–1399

Peyrard M, Pan HQ, Kedra D, Fransson I, Swahn S, Hartman K, Clifton SW, Roe BA and Dumanski JP (1996) Structure of the promoter and genomic organization of the human beta-adaptin gene (BAM22) from chromosome 22q12. *Genomics* **36**: 112–117

Rousseau-Merck MF, Versteege I, Legrand I, Couturier J, Mairal A, Delattre O and Aurias A (1999) hSNF5/INI1 inactivation is mainly associated with homozygous deletions and mitotic recombinations in rhabdold tumors. *Cancer Res* **59**: 3152–3156

Ruttledge MH, Sarrazin J, Rangaratnam S, Phelan CM, Twist E, Merel P, Delattre O, Thomas G, Nordenskjöld M, Collins VP, Dumanski JP and Rouleau GA (1994a) Evidence for the complete inactivation on the NF2 gene in the majority of sporadic meningiomas. *Nature Genetics* **6**: 180–184

Ruttledge MH, Xie Y-G, Han F-Y, Peyrard M, Collins VP, Nordenskjold M and Dumanski JP (1994b) Deletions on chromosome 22 in sporadic meningioma. *Genes Chrom Cancer* **10**: 122–130

Seizinger BR, Martuza RL and Gusella JF (1986) Loss of genes on chromosome 22 in tumorigenesis of human acoustic neuroma. *Nature* **322**: 644–647

Sevenet N, Lellouch-Tubiana A, Schofield D, Hoang-Xuan K, Gessler M, Birnbaum D, Jeanpierre C, Jouvet A and Delattre O (1999) Spectrum of hSNF5/INI1 somatic mutations in human cancer and genotype-phenotype correlations. *Hum Mol Genet* **8**: 2359–2368

Versteege I, Sevenet N, Lange J, Rousseau-Merck MF, Ambros P, Handgretinger R, Aurias A and Delattre O (1998) Truncating mutations of hSNF5/INI1 in aggressive paediatric cancer. *Nature* **394**: 203–206

von Deimling A, Bender B, Louis DN and Wiestler OD (1993) A rapid and non radioactive PCR based assay for the detection of allelic loss in human gliomas. *Neuropathology and Applied Neurobiology* **19**: 524–529

Wellenreuther R, Kraus J, Lenartz D, Menon AG, Schramm J, Louis DN, Ramesh V, Gusella JF, Wiestler OD and von Deimling A (1995) Analysis of the neurofibromatosis 2 gene reveals molecular variants of meningioma. *American Journal of Pathology* **146**: 827–832

© 2001 Cancer Research Campaign 

